# Preventive use of nitisinone in alkaptonuria

**DOI:** 10.1186/s13023-021-01977-0

**Published:** 2021-08-03

**Authors:** Bruce H. R. Wolffenbuttel, M. Rebecca Heiner-Fokkema, Francjan J. van Spronsen

**Affiliations:** 1 Department of Internal Medicine, Division of Endocrinology, University of Groningen, University Medical Center Groningen, P.O. Box 30001, 9700 RB Groningen, The Netherlands; 2 Department of Laboratory Medicine, University of Groningen, University Medical Center Groningen, Groningen, The Netherlands; 3 Beatrix Children’s Hospital, Division of Metabolic Disorders, University of Groningen, University Medical Center Groningen, Groningen, The Netherlands

**Keywords:** Alkaptonuria, Complications, Nitisinone, Personalized medicine, Prevention

## Abstract

Alkaptonuria (AKU, OMIM 203500) is a rare congenital disorder caused by a deficiency of the enzyme homogentisate-1,2,-dioxygenase. The long-term consequences of AKU are joint problems, cardiac valve abnormalities and renal problems. Landmark intervention studies with nitisinone 10 mg daily, suppressing an upstream enzyme activity, demonstrated its beneficial effects in AKU patients with established complications, which usually start to develop in the fourth decade. Lower dose of nitisinone in the range of 0.2–2 mg daily will already reduce urinary homogentisic acid (uHGA) excretion by > 90%, which may prevent AKU-related complications earlier in the course of the disease while limiting the possibility of side-effects related to the increase of plasma tyrosine levels caused by nitisinone. Future preventive studies should establish the lowest possible dose for an individual patient, the best age to start treatment and also collect evidence to which level uHGA excretion should be reduced to prevent complications.

## Introduction

**Fig. 1:**
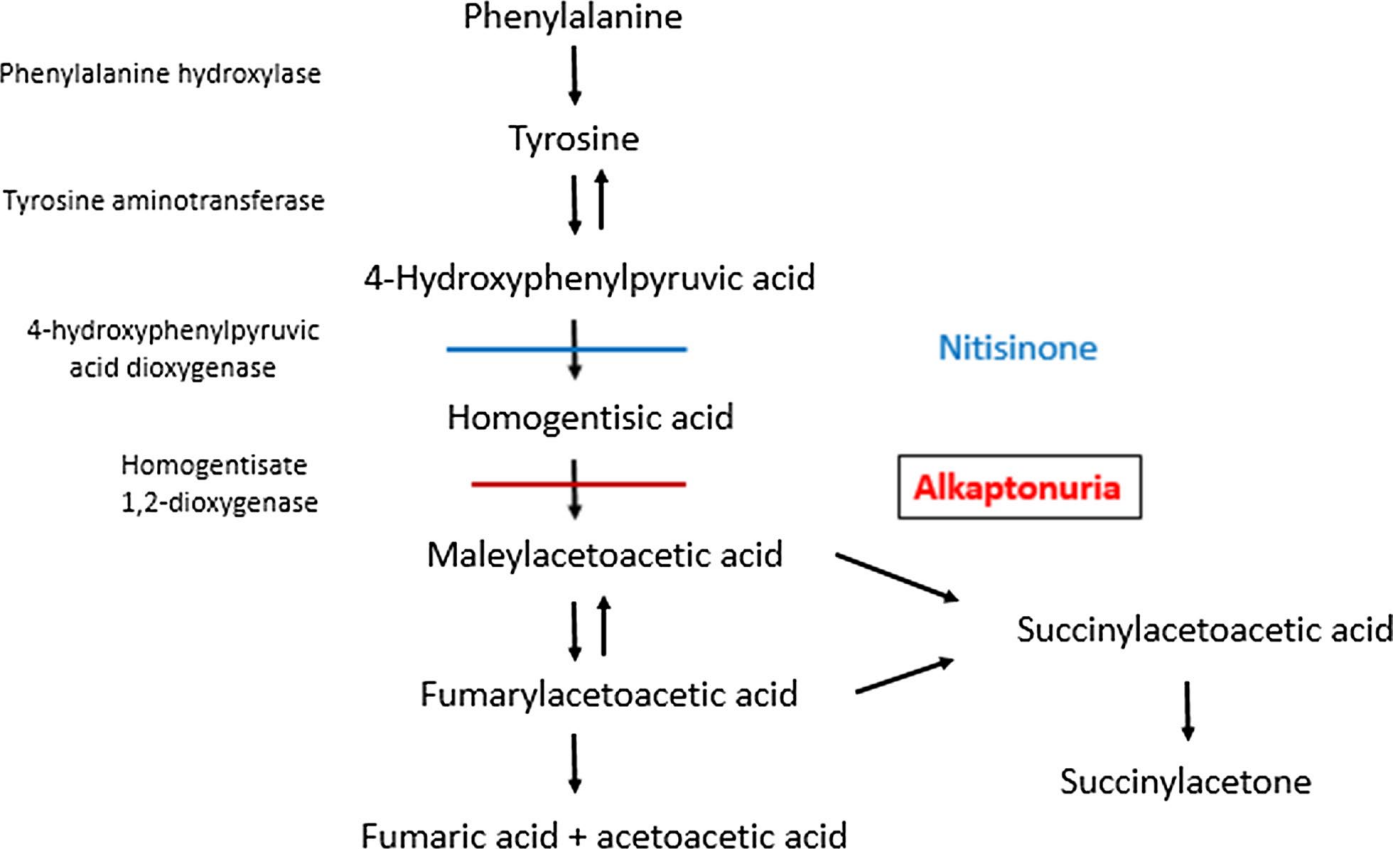
Metabolism of phenylalanine and tyrosine

Alkaptonuria (AKU, OMIM 203,500) is a congenital disorder caused by a deficiency of the enzyme homogentisate-1,2-dioxygenase (HGD), which catalyzes the conversion of homogentisic acid (HGA) to maleylacetoacetate. As a consequence there is an accumulation of HGA (Fig. 1) and its derivative benzoquinone-acetic acid in the body, which are toxic to several tissues like cartilage. The long-term consequences of AKU are joint problems, cardiac valve abnormalities, kidney stones and sometimes renal insufficiency [1]. Earlier studies have shown that the radiographic joint abnormalities start to develop after the age of 30 years, with the first joint replacement performed at a mean age of 55 years, while mean age of diagnosis of cardiac valve involvement is 54 years and coronary-artery calcification at 59 years [2]. The preventive effect of dietary protein restriction to influence the course of the disease has been limited [3].

## Recent advances in AKU treatment

Nitisinone (2-(2-nitro-4-(trifluoromethyl)benzoyl) cyclohexane-1,3-dione) is a drug which has been used for over 20 years for the treatment of children with Tyrosinaemia type I (TT1) [4]. It acts as an inhibitor of the enzyme 4-hydroxyphenyl pyruvic acid [5], and therefore can reduce the formation of HGA. Based on this action, it can be expected that treatment of AKU patients with nitisinone may therefore reduce the consequences of the disorder, especially the joint destruction and the ochronosis of for instance the eye which develops with long-standing disease. Indeed, several recent studies have shown that chronic treatment with 10 mg nitisinone daily for a period up to 4 years did reduce the rate of progression of AKU-related symptoms and arrest or even reverse ochronotic pigmentation [6]. This was confirmed in a larger study, SONIA-2, in which nitisinone 10 mg daily was compared to no treatment in 138 patients with AKU from the UK, France and Slovakia [7]. Nitisinone in this dose decreased urinary HGA by 99%, and after 4 years, the increase in AKU symptom score was statistically lower in the nitisinone-treated patients. Mean age of the participants at start of nitisinone was 48 years. Although it was mentioned that nitisinone was tolerated well, there was a higher incidence of infections and eye-related adverse events in the nitisinone-group. In total, 13% of patients stopped treatment with nitisinone due to adverse events, while dose reduction was advised in 12% of patients [7]. Ophthalmologic complications like keratopathy may however develop as a consequence of increasing tyrosine concentrations caused by the treatment [7] as especially seen in Tyrosinaemia type II, but also in patients with TT1 although the relation with blood tyrosine concentrations is not that clear.

## The clinical dilemma

These exciting results of nitisinone are now entering the discussions physicians are having with their AKU patients. The dilemmas discussed are considerable. Should we already commence treatment, and if so, what regimen should we advise to a 25-year old woman who is in excellent health without any clinical signs and symptoms of AKU-related complications. Would this be different from the treatment which we may propose to a 40-year old woman who is gradually experiencing back pain as a consequence of slowly-progressing spinal abnormalities, and a 60-year old man with severe clinical ochronosis who already underwent bilateral hip replacement and has moderate aortic valve sclerosis?

Although these landmark studies demonstrated the beneficial effects of nitisinone in AKU, we must realise that this therapy was started late in the course of the disease; most patients involved in the were between 40 and 60 years (mean age 52 years in the US National Institutes of Health (NIH) study [8], mean age 48 years in SONIA-2 [7]), and many already had suffered the consequences of the disease. The dose of nitisinone used in the former study was 2 mg daily [8], which was adapted in the latter study to 10 mg once daily, because of the fact that this dose lowers urinary HGA even slightly more, and the US NIH study did not report a significant beneficial effect on its primary and secondary parameters, i.e. change in motion of the worse-affected hip, and -amongst others- measurement of spinal flexion, 6-min walk times [7]. Based on the SONIA-2 results, the use of nitisinone (Orfadin^R^) 10 mg daily has been officially approved by the European Medicines Agency (EMA) in September 2020 for the treatment of adult patients with AKU [9]. The EMA had recommended to use physiological urinary levels of HGA as an endpoint. Nevertheless, there was no difference in the change of the clinical severity score between those with 24-h urinary HGA below and above 300 µmol. Most of the complications of AKU for joints and heart valves will be developing after the age of 30–40 years [1, 2], although several patients have been reported with signs of AKU, like ochronosis of eyes and ears, already in the second decade of life. The proven efficacy of nitisinone to mitigate or reverse the complications associated with AKU holds promise for early preventive therapy with this medication. There may be a large window of opportunity to start early treatment with nitisinone to reduce HGA levels in order to prevent such damage, and an observational study to assess the progression of AKU-related complications in children, the SOFIA-Paediatric study, is ongoing [10].

## Preventive treatment with low-dose nitisinone

In the situation of preventive medicine, there is a different balance between efficacy of a medical intervention, the acceptance of possible side-effects and costs of the intervention. The safer and better tolerated any treatment will be, the more likely physicians are willing to prescribe the drug and the more likely patients will be able to maintain this medication and benefit from the preventive effects. Nitisinone doses lower than those used in the recently published intervention studies may be helpful to prevent the development of complications, instead of combatting these once they are clinically evident. Early low-dose preventive treatment may postpone complications and prevent the necessity for joint replacements or cardiac valve repair or replacement, while in the same time there may be a lower risk of drug-related side-effects. It also necessitates a different approach to evaluate the clinical effects of the treatment. Several clinical scores and questionnaires have been used to evaluate the severity of AKU and impairment of health-related quality of life. The currently employed Alkaptonuria Severity Score Index (AKUSSI) is an excellent summary of already established complaints and complications [11], but can not be used for early disease monitoring. Questionnaires like the Knee injury and Osteoarthritis Outcome Score (KOOS) for pain, symptoms, daily living, sport and quality of life, the Hip disability and Osteoarthritis Outcome Score (HOOS), and the Quality of Life for Osteoporosis questionnaire (QUALEFFO-41), as well as the Health Assessment Questionnaire Disability Index (HAQ-DI), measuring limitations in daily life, and the global pain visual analogue scale (hapVAS) have been used and can be used to prospectively monitor patient’s complaints and quality of life [12].

Important questions such as the optimal age at start of treatment, the desired dose of nitisinone and the need of a diet need to be addressed in our conversations with patients [13]. When started early in the course of the disease, for instance in the third decade or even earlier, treatment with nitisinone—perhaps at even lower doses than used in the intervention studies—may be able to prevent the development of the long-term consequences as a result of its HGA-reducing properties. Indeed, in the SONIA-1 study, a dose-dependent reduction of HGA was reported after 4 weeks of treatment with nitisinone, which varied from a mean of 90% for the 1 mg dose to 98.8% for the 8 mg dose. Thus, the largest effect on HGA was already achieved with a rather low dose of nitisinone in adults. As urinary HGA excretion may vary considerably between participants, an approach in which an optimal reduction of urinary HGA concentration is aimed for with the lowest possible dose of nitisinone looks attractive, thereby preventing an exaggerated increase of plasma tyrosine levels and associated short-term and long-term side-effects in this otherwise symptom-free group of patients.

## Aiming for which HGA reduction?

**Fig. 2:**
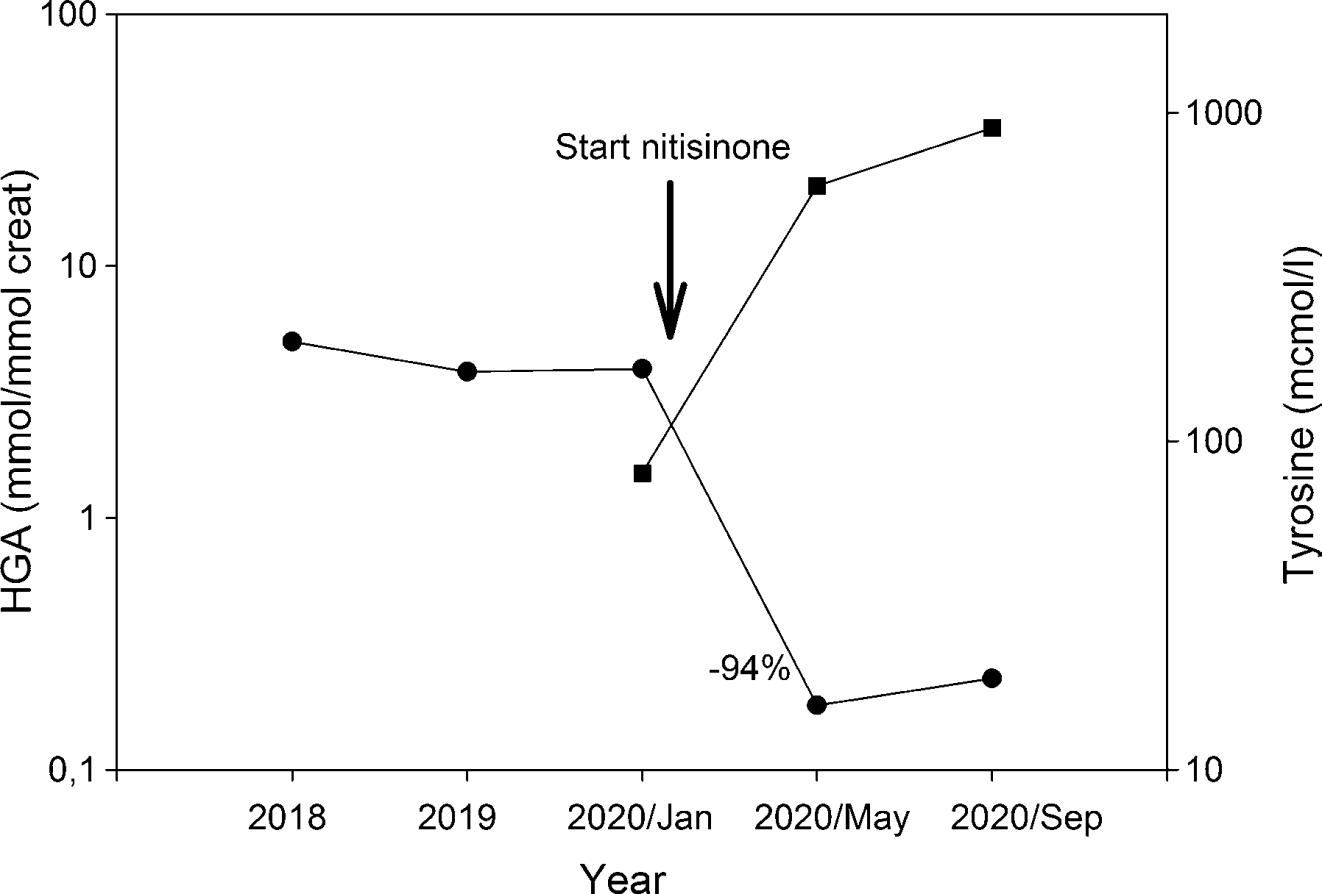
Course of urinary HGA concentration and serum tyrosine concentration after initiation of nitisinone therapy, 2 mg on alternate days

Although to date there is no known threshold below which tissue damage as a consequence of increased HGA concentrations does *not* occur, it can be postulated that any reduction of HGA levels early in the course of the disease may be beneficial in—at least—postponing the development of complications. In addition, HGA concentrations may differ greatly between patients [14], which also allows a personalized approach for reduction. Figure 2 shows the results of treatment with low-dose nitisinone in one of our patients. Nitisinone was initiated at a dose of 2 mg on alternating days. Four and eight months later, urinary HGA concentration was 0.18 and 0.23 mmol/mmol creatinine, respectively, reflecting a 94–95% reduction compared to pre-treatment levels. An increase of plasma tyrosine was observed to 600 and later 900 µmol/l, and, although the patient did not experience any complaints or side-effects of the medication, in a shared-decision making process it was decided that the dose of nitisinone was not further increased. As mentioned earlier, there was no difference in the change of the clinical severity score (cAKUSSI) between those with 24-h urinary HGA below and above 300 µmol, although those with higher urinary HGA were on average 10 years younger [9].

Several studies have assessed the effect of low dose nitisinone on urinary HGA excretion and plasma tyrosine levels (Table 1). One of the earlier studies exploring these effects in nine AKU patients reported a > 90% reduction of urinary HGA excretion with 1.05 mg bid, with an increase of plasma tyrosine level to 755 µmol/l [15]. Protein intake reduction in 5 of these patients led to a decrease of plasma tyrosine to 603 µmol/l [15]. A later study reported a > 95% reduction of urinary HGA excretion with 2 mg nitisinone daily in 40 AKU patients in the age range between 38 and 68 years, which effect was maintained throughout the follow-up of 36 months [8]. Plasma tyrosine levels averaged 800 µmol/l throughout the observation period without any protein restriction, and only two participants were reported to develop keratopathy. The effect of treatment on clinical symptoms was limited due to the fact that most of the participants had already significant AKU-related tissue damage [8]. In a more recent paper, doses of 0.2 mg per day were reported to lower HGA by > 90% in three AKU patients while plasma tyrosine levels were maintained below 500 µmol/l [16].

**Table 1:** Effect of low dose nitisinone on alkaptonuria-related variables

	N patients	Duration	Dose	Δ uHGA (%)	Mean pTyrosine on treatment (µmol/l)
Suwannarat et al. [15]	9	3–4 mo	1.05 mg bid	− 94	755 ± 167
					603 ± 114*
Introne et al. [8]	40	4 mo–3 yrs	2 mg od	− 95	670–826
Sloboda et al. [16]	3	3–5 yrs	0.2 mg od	− 90	305–416
Ranganath et al. [14]	8	4 wks	1 mg od	− 89.4	653 (450–806)
	8		2 mg od	− 94.9	715 (506–865)
	8		4 mg od	− 98.3	803 (657–1115)
	8		8 mg od	− 99	813 (523–927)

^*^After initiation of protein restriction

Bid, twice daily; mo, months; N, number; od, once daily; uHGA, urinary homogentisic acid excretion

## Side-effects related to nitisinone treatment

Main side effects of nitisinone treatment appear to be related to the increase of tyrosine levels, which amongst others may lead to keratopathy and eye complaints. This may necessitate cessation or a reduction of the nitisinone dose. On one hand it has been known that many AKU patients may develop high tyrosine levels without development of ocular complications [7]. On the other hand, patients may develop painless keratopathy even when treated with a low dose nitisinone [17]. In early animal studies it was noted that development of keratopathy was not simply related to plasma tyrosine levels, and did occur in beagle dogs but not in rhesus monkeys, possibly related to differences of tyrosine accumulation in the eye [7, 18].

Other side-effects of tyrosine which may warrant more attention are changes in cognitive functioning. From studies in another defect in the phenylalanine-tyrosine catabolic pathway (Fig. 1), Tyrosinaemia type II, it is known that the plasma level of tyrosine that could result in clinical manifestations is somewhere between 600 and 900 µmol/l. Nevertheless, some patients can tolerate plasma tyrosine levels over 1000 μmol/l without complications. From another defect in this cascade, TT1, it is known that increased tyrosine concentrations (as a consequence of nitisinone) may play a role in the risk of mental dysfunction in these patients [19, 20], while in younger patients low phenylalanine concentrations rather than high tyrosine concentrations seem to be more dangerous [21]. So, while in young AKU patients an earlier start of nitisinone (including a diet restricting the phenylalanine and tyrosine intake) may lead to an improved long-term functional outcome, at the same time, nitisinone treatment at least should be carefully used and monitored for development of mental and cognitive disturbances. Other studies have shown a dose-dependent decline of cognitive functioning in elderly patients with higher plasma levels of tyrosine, approaching 600 µmol/l during a 4-h infusion of tyrosine [22]. This finding may also be of relevance for the risk–benefit assessment of nitisinone treatment in elderly individuals with AKU.

## Unresolved questions and future studies

Thus, treatment with low dose nitisinone may be a game changer in the prevention of AKU-related complications, and allow an individual approach in young AKU patients [23]. Future studies are needed to address whether it is mandatory to reduce urinary HGA concentrations to those in normal individuals, and which other factors may determine the degree of residual harm caused by slightly elevated HGA concentrations. Earlier studies suggested to prevent the oxidation of HGA by the use of reducing agents such as ascorbic acid [24]. In the same time, protein intake with the diet should be monitored, and when protein restriction in order to reduce plasma tyrosine levels is started or strengthened, care should be taken to prevent micronutrient deficiencies like those of riboflavin, vitamin B12 and vitamin D. Also, more information on the long-term safety of the drug is needed, especially when started at an early age, i.e. in the second or third decade. Contra-indications for the use of nitisinone are limited: any treatment with nitisinone should be interrupted in women intending to become pregnant or who are breastfeeding, although uneventful pregnancy with a low dose of nitisinone has been reported [16]. Yearly costs of treatment in The Netherlands are approximately € 2000 for the prescription of 2 mg nitisinone on alternate days, and € 14,300 for the 10 mg daily dose. Although many questions need to be resolved in future studies, this should however not deter clinicians from discussing with their AKU patients in a shared-decision making process when and how to start early treatment in order to prevent the severe long-term consequences of this disorder.

In summary, several recently published hallmark studies show the benefit of treatment of AKU patients with nitisinone. Together with the knowledge that low doses of the drug may already lower HGA concentrations by > 90%, this offers a much-awaited possibility to improve the long-term perspectives of these patients.

## Data Availability

Not applicable.

## Abbreviations

AKU: Alkaptonuria
AKUSSI: Alkaptonuria Severity Score Index
EMA: European Medicines Agency
hapVAS: Global pain visual analog scale
HAQ-DI: Health Assessment Questionnaire Disability Index
HGA: Homogentisic acid
HGD: Homogentisate-1,2,-dioxygenase
HOOS: Hip disability and Osteoarthritis Outcome Score (HOOS)
KOOS: Knee injury and Osteoarthritis Outcome Score
NIH: US National Institutes of Health
QUALEFFO-41: Quality of Life for Osteoporosis questionnaire
uHGA: Urinary homogentisic acid excretion
